# Framework and components for effective discharge planning system: a delphi methodology

**DOI:** 10.1186/1472-6963-12-396

**Published:** 2012-11-14

**Authors:** Carrie HK Yam, Eliza LY Wong, Annie WL Cheung, Frank WK Chan, Fiona YY Wong, Eng-kiong Yeoh

**Affiliations:** 1 Division of Health System, Policy and Management, JC School of Public Health and Primary Care, The Chinese University of Hong Kong, Hong Kong, Hong Kong

## Abstract

**Background:**

To reduce avoidable hospital readmissions, effective discharge planning and appropriate post discharge support care are key requirements. This study is a 3-staged process to develop, pretest and pilot a framework for an effective discharge planning system in Hong Kong. This paper reports on the methodology of Delphi approach and findings of the second stage on pre-testing the framework developed so as to validate and attest to its applicability and practicability in which consensus was sought on the key components of discharge planning.

**Methods:**

Delphi methodology was adopted to engage a group of experienced healthcare professionals to rate and discuss the framework and components of an effective discharge planning. The framework was consisted 36 statements under 5 major themes: initial screening, discharge planning process, coordination of discharge, implementation of discharge, and post discharge follow-up. Each statement was rated independently based on 3 aspects including clarity, validity and applicability on a 5-point Likert-scale. Statement with 75% or above of participants scoring 4–5 on all 3 aspects would be included in the discharge planning framework. For those statements not reaching 75% of consensus in any one of the aspect, it would be revised or discarded following the group discussion, and be re-rated in another round.

**Results:**

A total of 24 participants participated in the consensus-building process. In round one rating, consensus was achieved in 25 out of 36 statements. Among those 11 statements not reaching consensus, the major concern was related to the “applicability” of the statements. The participants expressed a lack of manpower, skills and time in particular during weekends and long holidays in carrying out assessment and care plans within 24 h after admission. There were also timeliness and availability issue in providing transportation and necessary equipment to the patients. To make the statements more applicable, the wordings of some of the statements were revised to provide greater flexibility. Due to the lack of a statement in clarifying the role of the members of the healthcare professional team, one additional statement on the role and responsibility of the multidisciplinary team members was added. The first theme on “initial screening” was further revised to “initial screening and assessment” to better reflect the first stage of discharge planning process. After two rounds of rating process, all the 36 statements and the newly added statement reached consensus

**Conclusions:**

A structured, systematic and coordinated system of hospital discharge system is required to facilitate the discharge process to ensure a smooth patient transition from the hospital to the community and improve patient health outcome in both clinical and social aspect. The findings of this paper provide a reference framework helping policymakers and hospital managers to facilitate the development of a coherent and systematized discharge planning process. Adopting a Delphi approach also demonstrates the values of the method as a pre-test (before the clinical run) of the components and requirements of a discharge planning system taking into account of the local context and system constraints, which would lead to improvements to its applicability and practicability. To confirm the applicability and practicability of this consensus framework for discharge planning system, the third stage of process of development of the discharge planning framework is to apply and pilot the framework in a hospital setting to evaluate its feasibility, applicability and impact in hospital including satisfaction from both the perspectives of staff and patients.

## Background

Hospital readmission rate has been gained increasingly attention because it reflects the effectiveness of healthcare system performance and the quality of patient care [1-4]. A number of studies highlighted that an effective discharge planning is crucial to improve continuity of care between hospital and home/elderly home so as to improve patient’s health and reduce patient readmission [4-9]. Discharge planning in hospital is pivotal in the continuing care of people who are in need of medical, social and rehabilitation care [7,10]. As the needs of patients have increased and become more complex, it is also important that an effective discharge planning system should have the capacity to discriminate and respond to different levels of need for coordination and post-discharge care [11]. In particular, “continuity management” plays an important role especially in chronic or complex clinical diseases that require management from multidisciplinary team [7]. Therefore, a well defined discharge planning system is crucial in facilitating management continuity and providing predictability and security in future care for both patients and providers [7].

In view of the importance of an effective discharge planning system, many countries have launched a series of guidelines or policy-driven frameworks for good practices in hospital discharge planning processes. In general, discharge planning is conceptualized as having four phases: (1) patient assessment; (2) development of a discharge plan; (3) provision of service, including patient/family education and service referral; and (4) follow-up/evaluation [12]. The components of discharge planning system might vary in different countries because of the differences in healthcare systems, how health and social services are organized, and nature of patient’s post-discharge needs and cultural concerns. In Australia, the Victoria Government has identified four important components in the “Effective Discharge Strategy” to all Victorian public hospitals [11]. The four components included (i) assessment of patient’s physiological, psychological, social and cultural needs; (ii) care plan development by identifying discharge strategies to patient, carer, and community provider; (iii) implementation of the plan including information delivered, education provided, and (iv) coordination of services. In United States, discharge planning is a legally mandated function for hospitals as outlined in Medicare’s Conditions of Participation from Centres for Medicare & Medicaid Services. Its purposes are to identify patients who suffer adverse consequences, collaboratively determine the proper care level with appropriate healthcare professionals, match patients to the most appropriate post-acute services, and assure a smooth, planned and gap free transition of patients to the next level of care [13]. In United Kingdom, the National Health Services have initiated discharge planning system upon patient admission so as to triage the simple or complex discharge which is then followed by a series detailed assessment, planning and delivery by multi-disciplinary team and multi-agency working [14,15]. NHS further highlighted a policy framework with emphasis on the development of timely simple discharge and agreement on more specific guidance and criteria for different patient groups [16].

In Hong Kong, the discharge planning is conducted on a piecemeal basis in different specialties and hospitals, and often lacks coordination and input from the different healthcare disciplines. There is also a rapid turnover of large numbers of patients with acute (and often undifferentiated) illness in hospitals that presents challenge for a structured discharge planning system. In addition, our previous study on avoidable readmission showed that around 40% of hospital readmissions in Hong Kong could have been prevented, and the avoidable readmissions were intimately related to clinical management and patient care [17]. This study highlighted the need for an effective discharge planning system to improve current discharge processes and facilitate the continuity of patient management. Another study in Hong Kong also echoed the need for the development of a comprehensive, system-wide, and policy-driven discharge planning process [6]. It also emphasized the importance of better communication and coordination across different service providers and patients in both acute and sub-acute care provisions.

In building a comprehensive discharge planning system, a framework for systems-wide discharge planning is needed to identify the differing needs of the varied patients and what is appropriate for them within the context of the services available. Some authors such as LeClerc and Wells used process evaluation to develop and evaluate the discharge planning model for acutely-ill elderly patients in Canada [18]. Firstly, they identified seven key elements of discharge planning including the designation of a single discharge manager, the inclusion of the physician as a core participant, and the presence of well-working communications systems using a qualitative study among patients and professionals [19]. These seven key elements were then translated and operatioanlized into a protocol which outlined the timing and process of discharge-related interventions. A process evaluation was then conducted to evaluate this integrated model of discharge planning using case study design [18]. The feasibility of implementing the discharge planning model and the corresponding facilitators and challenges were obtained using the explanation-building and case comparison method among the four case studies of the discharge planning performed by the discharge manager in the hospitals.

Delphi study is a structured group communication process/technique which is commonly used to seek a consensus of opinions on a specific topic among homogeneous groups of experts [20]. The Delphi process has been used in various fields including social policy and public health [21]. Recently, it has been used in achieving an international consensus on hospital discharge criteria for patients undergoing colorectal surgery [22]. The Delphi process has also been applied to reach consensus among occupational therapists on the multidisciplinary teamwork in discharge planning [23].

**Figure 1:**
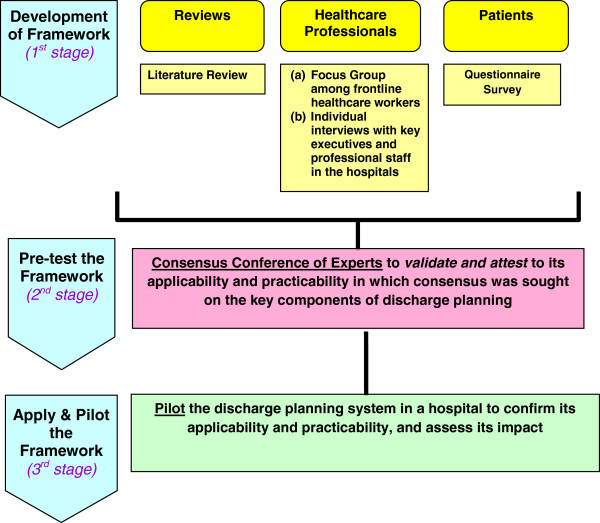
A 3-staged approach to develop a framework for an effective discharge planning system in Hong Kong.

Our study adopted a 3-staged approach to develop a framework for an effective discharge planning system in Hong Kong (Figure 1). In the first stage of development, the identification of the components for the framework was drawn by (i) taking reference of international experiences such as UK and Australia [11,16], and (ii) collecting different stakeholders’ views on an effective discharge planning system through (a) telephone interviews among discharge patients [24] to assess patient’s engagement in discharge planning process and their post discharge care needs, (b) focus group discussions of frontline healthcare workers to explore the barriers to discharge planning [6] and (c) individual interviews with key executives and professional staff in the hospitals. The findings of the literature review and focus groups provided the basis in developing the key themes of discharge planning framework. The key themes were then translated into statements by using the results of interviews and focus groups. The next stage in the development of this framework was to apply the method of Delphi to pre-test the initial framework developed. The objective of this step was to pre-test the framework to validate and attest to its applicability and practicability at an expert conference in which consensus was sought on the key components of discharge planning. The third stage which was in progress was to apply and pilot the discharge planning framework arrived at the consensus conference of experts, in a hospital to confirm its applicability and practicability, and assess its impact. This paper reported on the methodology and findings of the second stage using the consensus conference of experts in pre-testing the framework for an effective discharge planning system.

## Methods

### Consensus conference of experts

A consensus conference of experts was held to validate the discharge planning framework developed in the first stage and attest to its applicability and practicability. In developing the consensus framework for an effective discharge planning, the Delphi methodology - a systematic approach to engage a large number of experts in a process to derive consensus in a group by rating the research framework- was adopted in this study. The characteristics of a Delphi study include anonymity, iteration, controlled feedback and statistical group response which allow the participants to provide and change their opinion freely [25,26]. Since the frontline practical experience is important in developing the framework for discharge planning, participants were engaged to discuss how to revise the framework between each round of rating. To maintain an independent rating, discussion among participants during rating for the amended statements was not allowed.

### Participants

A study suggested that Delphi respondent sample size of above 15 participants yields little improvement in reliability due to diminishing returns [27]. In consideration of obtaining views from different healthcare professionals who involve in the discharge planning process, we targeted at recruiting 3–4 participants from each of the following key members of multidisciplinary healthcare team including physicians, nurses, physiotherapists, occupational therapists and medical social workers. There were no age and sex exclusions for study entry. However, to ensure the professionals’ qualifications and clinical expertise, participants were required to have at least 5 years working experience in the health-related field; currently work in the Medical departments of acute or rehabilitation hospitals/community service provision; have knowledge and understanding of the discharge planning system; and have commitment to contribute to each round of rating.

### Instrument

The questionnaire consisted of 36 statements under 5 major themes of discharge planning framework: (i) initial screening, (ii) discharge planning process, (iii) coordination of discharge, (iv) implementation of discharge, and (v) post discharge follow-up (Additional file 1). The themes were developed on the basis of a literature review which included 210 relevant articles identified from the Medline database and Internet using “discharge planning” and “post discharge care” as keywords; and the findings of focus group discussions among frontline healthcare workers. The statements were then drawn up from the findings of the questionnaire survey with discharge patients & their caregivers and individual interviews with key corporate executives and healthcare professionals in the hospitals, and focus groups with frontline healthcare workers [6,24]. In particular, the literature review provided the basic key components of discharge planning and the potential initial screening items that could be used upon hospital admission. The patient questionnaire survey highlighted the importance of timely identification of patients’ needs and provision of adequate follow-up support in discharge planning in a local context [24]. The focus groups, on the other hand, provided important views on the barriers that could be experienced in hospital discharge with regard to factor of system, healthcare professionals, patients, and social [6]. The findings suggested the importance of clearly identified staff roles and better communication and coordination across the various health and social care providers.

### Procedures to achieve consensus

A one-day consensus conference of experts was held on 24 August 2011 to develop the consensus framework for an effective discharge planning. The participants received background reading materials including a report of the literature review of discharge planning and the proposed themes and statements a week before the conference. Each participant was given guidelines which explained the purpose of study and the steps to complete the questionnaire. The participants were also briefed on the background of the research and the proposed framework for discharge planning.

A self-administered questionnaire was used to rate the components of the proposed framework. Participants were requested to rate each statement independently in three aspects: clarity, validity and applicability on a 5-point Likert-scale based on their views and experience. The ratings were grouped into three levels – low score (rating 1–2), average score (rating 3) and high score (rating 4–5). Clarity was defined as the extent to which the statement was expressed in clear, precise and unambiguous language. Validity was defined as the extent to which the statement was relevant for the development of an effective discharge planning system. Applicability was defined as the extent to which the statement was appropriate to be applied in the discharge planning system. A study in evaluating the methods of Delphi study suggested a 75% level of consensus of agreement [25]. Therefore, for those statements not reaching 75% of consensus for a high score (rating 4–5) in any one of the aspect would be revised or discarded following the group discussion, and be re-rated in another round in the study. The number of round of ratings to achieve consensus depended on the level of consensus reached in each statement.

### Data analysis

Descriptive statistics for each statement in the questionnaire were calculated to check whether the raw score of each statement reaches 75% of consensus in clarity, validity and applicability. Median with inter-quartile range (IQR) was also chosen to report the degree of consensus. Median was chosen because the distribution of ratings was generally skewed and a visual check confirmed that none were bimodal [28]. Median ranging from 1–5 was used to indicate the degree of support from the participants for each statement where a higher median suggested a greater degree of consensus. In addition, degree of consensus can be derived from measuring the spread of responses by looking at the inter-quartile range (IQR), generated by taking the difference between the 25^th^ and 75^th^ percentiles where larger IQR indicates greater divergence in opinion. Kruskal-Wallis H tests were further performed to test the differences in the mean ratings of different healthcare professions. The level of statistical significance was set at p-value <0.05.

### Ethical consideration

Ethical approval was obtained for the study from the Hong Kong Hospital Authority Kowloon West Cluster Clinical Research Ethics Committee.

## Results

### Participants

A total of 24 participants participated in the consensus process including five medical doctors, six nurses, five physiotherapists, four occupational therapists, and four medical social workers. 71% of participants were female with a mean age of 46 (standard deviation: 7 years). Most of them (67%) had 20–29 years of working experience (mean: 23 years; standard deviation: 7 years). Details of their demographics are shown in Table 1. A 100% response rate was obtained in study since all participants were gathered in a conference to rate the statements.

**Table 1:** Demographics of participants (N=24)

	**n**	**%**
**Gender**		
Female	17	70.8
Male	7	29.2
**Age**		
20-29	1	4.2
30-39	2	8.3
40-49	14	58.3
50-59	7	29.2
**Type of healthcare professions**		
Doctor	5	20.8
Nurse	6	25.0
Physiotherapist	5	20.8
Occupational therapist	4	16.7
Medical social worker	4	16.7
**Year of working experience**		
<10	1	4.2
10-19	5	20.8
20-29	16	66.7
30-39	2	8.3

### Round 1 rating results

In round one, a consensus was achieved in 25 out of 36 statements with at least 75% of respondents rating 4–5 on clarity, validity and applicability in these 25 statements. Eleven statements did not reach consensus (Table 2). One statement on screening tool (Q1b) did not reach consensus on the “validity” aspect, with only 67% rating 4–5 on validity. Nine statements mainly relating to the timeliness and availability issues, in performing risk screening and care plan, and providing transportation and necessary equipment to the patients (Q1a, Q1f, Q2e, Q3c,Q3f, Q3g, Q4d, Q4h, Q4j) did not reach consensus on the “applicability” aspect. One statement on the conduct of care plan within 24 h after admission (Q2b) did not reach consensus on both “validity” (71% rating 4–5), and “applicability” (29% rating 4–5) aspects. All the statements reached consensus on “clarity” aspect.

**Table 2:** Clarity, Validity and Applicability of those 11 sentences which do not reach consensus

**No.**	**Statement**	**% of respondents rated 4–5 on a 5-point scale**		
		**Clarity**	**Validity**	**Applicability**
	**Theme 1: Initial screening**			
1a	An initial risk screening should be performed within 24 h after admission to identify those patients with high risk of admission and have complex discharge planning, required to provide ongoing care and additional support after leaving hospital.	96	88	***42***
1b	HARRPE (Hospital Admissions Risk Reduction Program for the elderly), a screening tool developed by HA, could be used to stratify those elderly aged 60 or above with a higher risk of hospital readmission.	96	***67***	79
1f	The following items should be included in the initial assessment for all patients to serve as flags to trigger discharge planning as appropriate: Any change of ADL: ADL Barthel Index before admission, and on admission (declining ADL index)	83	88	***67***
	**Theme 2: Discharge planning process: including ongoing clinical and functional assessment to facilitate the development of care plan and final discharge plan**			
2b	Care plan should be performed within 24 h after admission.	92	***71***	***29***
2e	Systems for the accurate and timely communication of assessment and associated care planning information across clinical disciplines and settings should be developed and implemented to enhance care continuity.	88	100	***71***
	**Theme 3: Coordination of discharge: continuing and timely process from hospital stay to discharge**			
3c	Case conference should be considered for high risk patients for better communication between team members in the multidisciplinary team and to enable seamless and timely transition from hospital to community.	88	83	***71***
3f	Formal mechanisms for information transfer across clinical and social settings e.g. through discharge summary should be adopted rather than solely relying on informal communication between health and social professionals.	92	92	***67***
3g	Prompt provision of all community equipment including walking aids, wheelchairs, low vision or hearing aids, safety alarm, urinal, blood pressure machines, glucometers, visual door etc. should be ensured before discharge.	88	83	***46***
	**Theme 4: Implementation of discharge**			
4d	A patient copy of discharge summary and/or nursing discharge summary should be given to patients/carers on the date of discharge.	100	83	***63***
4h	When transport is to be used, this should be booked at least 24 h, where feasible, in advance of discharge.	96	96	***67***
4j	A “Patient Checklist” should be completed by the patient or carers before discharge to ensure that they understand the discharge plan and their needs are addressed.	100	92	***58***

With regard to the analysis by median and IQR, out of 36 statements, only the applicability aspect of 3 statements had a median of 3 in the range from 1–5, all others were 4 or above. These 3 statements were concerned with initial risk screening and care plan to be done within 24 h of admission respectively (Q1a & 2b), and prompt provision of all community equipment (Q3g). Also, the majority of the statements (31 statements) had a high degree of consensus within the group with IQR of 1 or below. Only 5 statements had a IQR of 1.75: 4 statements were related to the “clarity” aspect *(Q1e, 1h, 1i, 1j concerning with using care support, history of fall risk, mental state and medications respectively as the initial assessment items)* and 1 statement relating to the “applicability” aspect (*Q3c: Case conference should be considered for high risk patients*) Descriptive statistics including median, IQR, mean and standard deviation (SD) is shown in Table 3.

**Table 3:** Descriptive statistics of round one rating (36 statements)

	Clarity*				Validity*				Applicability*				**Remark**
	**Median**	**IQR**	**Mean**	**SD**	**Median**	**IQR**	**Mean**	**SD**	**Median**	**IQR**	**Mean**	**SD**	
**Theme 1: Initial screening**													
1a. An initial risk screening should be performed within 24 h after admission to identify those patients with high risk of admission and have complex discharge planning, required to provide ongoing care and additional support after leaving hospital.	4.00	1.00	4.33	0.70	4.00	0.00	3.96	0.75	3.00	1.00	3.38	0.71	Discussion ^1^ (applicability issue)
1b. HARRPE (Hospital Admissions Risk Reduction Program for the elderly), a screening tool developed by HA, could be used to stratify those elderly aged 60 or above with a higher risk of hospital readmission.	4.00	1.00	4.33	0.57	4.00	1.00	3.83	0.82	4.00	0.00	3.96	0.62	Discussion ^2^ (validity issue)
1c. A patient with score of above 0.2 is considered as high risk and requires a complex discharge arrangement.	4.00	1.00	4.43	0.59	4.00	1.00	4.17	0.58	4.00	0.00	4.13	0.46	
The following items should be included in the initial assessment for all patients to serve as flags to trigger discharge planning as appropriate:													
1d. Social support – living alone, day time alone, night time alone, with maid, with spouse, with children, with grandchildren, with others.	4.5	1	4.08	1.21	4	1	4.17	0.82	4	1	4.29	0.69	
1e. Care support – Yes (by spouse, son, daughter-in-law, daughter, son-in-law, grandchildren, maid, others), No	4	1.75	4.08	1.06	4	1	4.25	0.68	4	1	4.25	0.79	
1f. Any change of ADL: ADL Barthel Index before admission, and on admission (declining ADL index)	4	1	4.21	0.72	4	1	4.08	0.88	4	1	3.71	1.08	Discussion ^3^ (applicability issue)
1g. Functional ambulatory category (modified): lyer, sitter, dependent walker, assisted walker, supervised walker, indoor walker, outdoor walker (independent, assisted with carer, assisted with equipment)	4	1	4.21	0.66	4	1	4.25	0.79	4	1	4.25	0.90	
1h. History of fall risk for the past one year: No history of fall, history of fall = 1, recurrent falls, present to medical attention for fall, both risk factors are present	4	1.75	4.00	0.93	4	0.75	4.21	0.51	4	0	4.13	0.54	
1i. Mental state: normal, disorientated, disturbed, poor memory, not communicate	4	1.75	4.00	0.93	4	1	4.25	0.53	4	0.75	4.00	0.78	
1j. Medications: good drug compliance, poor drug compliance	4	1.75	4.13	0.90	4	1	4.33	0.57	4	0.75	4.04	0.69	
**Theme 2: Discharge planning process**													
2a. The four main dimensions for assessment should include medical health, physical, psychological and social functioning.	5	1	4.63	0.50	5	1	4.58	0.50	4	1	4.21	0.66	
2b. Care plan should be performed within 24 h after admission.	5	1	4.46	0.66	4	1	3.83	0.87	3	1	3.08	0.72	Discussion ^4^ (validity & applicability issue)
2c. Three categories of discharge plans could be developed based on the complexity of patients and assessment of their needs:	4	1	4.41	0.59	4	1	4.32	0.48	4	0	4.00	0.44	
● Generic discharge plan suitable for simple cases													
●Disease-based discharge plan suitable for complex cases when there are disease specific protocols													
●Non-disease specific, but tailored, discharge plan for complex cases identifying either by HARRPE or by assessment													
2d. Ongoing assessment/evaluation should be conducted throughout the episode of care to review and update the conditions of patients.	5	1	4.70	0.47	5	1	4.61	0.50	4	0	3.96	0.64	
2e. Systems for the accurate and timely communication of assessment and associated care planning information across clinical disciplines and settings should be developed and implemented to enhance care continuity.	4	1	4.29	0.69	4.5	1	4.50	0.51	4	1	3.79	0.59	Discussion ^5^ (applicability issue)
**Theme 3: Coordination of discharge**													
3a. A designated person e.g. a designated doctor, nurse, or allied health professional should be notionally responsible for ensuring that all aspects of discharge planning have been addressed by the time of discharge.	5	1	4.54	0.51	5	1	4.50	0.59	4	0	3.88	0.54	
3b. Once the patient is identified to have complex care needs, the designated person should initiate discharge planning with a multidisciplinary approach.	4.5	1	4.46	0.59	4	1	4.33	0.64	4	0	4.00	0.59	
3c. Case conference should be considered for high risk patients for better communication between team members in the multidisciplinary team and to enable seamless and timely transition from hospital to community.	5	1	4.46	0.72	4	1	4.29	0.75	4	1.75	3.96	0.75	Discussion ^6^ (applicability issue)
3d. The suitability of discharge destination e.g. whether home or old-aged home should be assessed to ascertain whether the support required is available.	4	1	4.25	0.74	4	1	4.25	0.61	4	0	3.88	0.54	
3e. Referral/arrangement for social support services should be initiated once the patient is assessed to have post discharge support need in the community.	5	1	4.42	0.78	5	1	4.50	0.59	4	0	3.88	0.80	
3f. Formal mechanisms for information transfer across clinical and social settings e.g. through discharge summary should be adopted rather than solely relying on informal communication between health and social professionals.	4	1	4.29	0.91	4	1	4.25	0.74	4	1	3.75	0.74	Discussion ^7^ (applicability issue)
3g. Prompt provision of all community equipment including walking aids, wheelchairs, low vision or hearing aids, safety alarm, urinal, blood pressure machines, glucometers, visual door etc. should be ensured before discharge.	4.5	1	4.38	0.71	4	1	4.04	0.86	3	1	3.54	0.88	Discussion ^8^ (applicability issue)
3h. Appropriate education and training should be provided to patients/carers to ensure that they understand how to use the equipment.	5	1	4.5	0.66	4	1	4.42	0.58	4	0	4.13	0.54	
3i. Appropriate information and education on medication management including side effects of medication should be provided to patients/carers before discharge.	5	1	4.54	0.51	5	1	4.54	0.51	4	0	4.04	0.62	
**Theme 4: Implementation of discharge**													
4a. Patients and/or carers should be engaged in the preparation of the discharge process.	5	1	4.71	0.46	5	1	4.67	0.48	4	0	3.92	0.65	
4b. Appropriate information on their illness should be given to the patients/carers to ensure that they could manage their ongoing care after discharge.	5	1	4.58	0.58	4.5	1	4.46	0.59	4	0	4.08	0.50	
4c. Patients/carers should be informed of any danger signals they should be aware of before discharge.	5	1	4.63	0.58	5	1	4.54	0.59	4	0	4.13	0.54	
4d. A patient copy of discharge summary and/or nursing discharge summary should be given to patients/carers on the date of discharge.	5	1	4.54	0.51	4	1	4.13	0.68	4	1	3.5	0.79	Discussion ^9^ (applicability issue)
4e. If the patient has complex care needs/disease specific problem, a contact information should be provided on who to contact if they are concerned about their condition or treatment after discharge.	4	1	4.38	0.71	4	1	4.38	0.58	4	0.75	3.88	0.74	
4f. Discharge summaries with necessary information should be issued to the facilities or care providers e.g. old aged homes within 48 h of discharge.	4.5	1	4.42	0.65	4	1	4.17	0.87	4	0.75	3.92	0.78	
4g. Discharge summaries with necessary information should be issued to the Hospital Authority outpatient and day care services within a week of discharge.	4.5	1	4.38	0.77	4	1	4.21	0.88	4	1	4.08	0.88	
4h. When transport is to be used, this should be booked at least 24 h, where feasible, in advance of discharge.	5	1	4.46	0.72	4	1	4.33	0.70	4	1	3.75	0.85	Discussion ^10^ (applicability issue)
4i. Timely transport arrangements when attending outpatient appointments should be made if necessary.	4	1	4.29	0.86	4	1	4.21	0.72	4	0.75	3.79	0.78	
4j. A “Patient Checklist” should be completed by the patient or carers before discharge to ensure that they understand the discharge plan and their needs are addressed.	4	1	4.46	0.51	4	0.75	4.17	0.57	4	1	3.5	0.93	Discussion ^11^ (applicability issue)
**Theme 5: Post discharge follow up**													
5a. If the patient has complex care needs and is transferred from an acute hospital to a rehabilitation hospital, verbal communication via telephone or written information about the patient’s conditions should be made between the healthcare professionals in acute and rehabilitation hospitals.	5	1	4.46	0.78	4.5	1	4.46	0.59	4	1	4.25	0.68	
5b. If the patient is referred to disease specific or special discharge programmes, person-to-person communication or written information about the patient’s conditions should be made between different parties.	4	1	4.29	0.81	4	1	4.38	0.58	4	1	4.17	0.64	

* Clarity, validity and applicability were rated in a 1–5 scale: 1–2 (low), 3 (average), 4–5 (high).

### Group discussion of statements not reaching consensus

Eleven statements which did not reach the consensus of 75% were brought to group discussion before the second round of rating. In the discussion, 24 participants were divided into three groups consisting of different healthcare professionals to revise the 11 statements which did not reach consensus (around 3–5 statements for each group). The revision was based on discussions of the feedback from the respondents written in the questionnaire, facilitated by the investigators of this study. Participants’ main concern on these statements was mainly related to operational concerns, including tools required and manpower management, in particular during weekends and long holidays in carrying out the assessment and care plan within 24 h after admission. There were also timeliness and availability issues, in providing transportation and necessary equipment to the patients. To make the statements more applicable, the wordings of some of the statements were revised to provide greater flexibility, for example, from “performed” to “initiated”, “ensured” to “facilitated”, “booked at least 24 h” to “timely transport arrangement”, etc. to reflect practicability consideration. In addition, with regard to the cooperation and communication across clinical disciplines and settings, participants agreed it was an important component in an effective discharge planning; however, it depended on the resources and timeliness of multidisciplinary input. Participants expressed that effective and accessible IT systems for communication across clinical disciplines and settings were essential to facilitate the timely communication. Formal mechanisms for information transfer to community services providers were also required, however, patients’ privacy issues have to be addressed.

Some participants further indicated that there was no statement in clarifying the role of the members of the healthcare professional teams. Therefore, one additional statement *“The role and responsibility of different healthcare professionals for the different tasks in the discharge planning process should be clarified”* was added for round two rating. Thus, a total of 12 statements were brought to second round of rating.

### Round 2 rating results

In round two, consensus was achieved on all the revised 11 statements and the additional statement, that was 75% or more of the respondents rated them 4–5 on all aspects. All statements had a median of 4 or above, and IQR was 1 or below, suggesting a high degree of consensus within the group. However, the clarity aspect of the revised statement “*Q3f: Relevant information should be available to related clinical and social settings.”* was decreased from 92% to 75%. The participants thought it was unclear on the meaning of “relevant information” and “clinical and social setting”, thus the statement was further revised as *“Formal mechanisms for information transfer to community services providers for continuity of care should be established”*. All the participants re-rated 4 or above on clarity, validity and applicability aspect of this further revised statement upon the completion of second round rating. The consensus-based framework for an effective discharge planning was, therefore, achieved after two rounds of the rating process.

Upon the agreement on all statements, some participants suggested fine-tuning the title of first theme (revised from “initial screening” to “initial screening and assessment”) to better reflect the first stage of discharge planning process. Table 4 showed all the statements under corresponding themes in which consensus were achieved.

**Table 4:** The consensus framework for an effective discharge planning system

**Theme 1: Initial screening & assessment**	**Remarks**
**Initial screening:**	
**1a:** An initial risk screening should be performed within 24 h after admission to differentiate patients with simple or complex discharge planning needs.	Modified
**1b:** HARRPE (Hospital Admissions Risk Reduction Program for the elderly) is one of the screening tools which could be used to identify a proportion of elderly aged 60 or above with a higher risk of hospital readmission.	Modified
**1c:** A patient with score of above 0.2 is considered as high risk and requires a complex discharge arrangement.	
**Assessment:**	
The following items should be included in the initial assessment for all patients to serve as flags to trigger discharge planning as appropriate:	
**1d:** Social support – living alone, day time alone, night time alone, with maid, with spouse, with children, with grandchildren, with others.	
**1e:** Care support – Yes (by spouse, son, daughter-in-law, daughter, son-in-law, grandchildren, maid, others), No	
**1f:** Any change of ADL on admission compared with pre-morbid state before this admission e.g. change of Barthel Index if	Modified
available	
**1g:** Functional ambulatory category (modified): lyer, sitter, dependent walker, assisted walker, supervised walker, indoor walker,	
outdoor walker (independent, assisted with carer, assisted with equipment)	
**1h:** History of fall risk for the past one year: No history of fall, history of fall = 1, recurrent falls, present to medical attention for fall, both risk factors are present	
**1i:** Mental state: normal, disorientated, disturbed, poor memory, not communicate	
**1j:** Medications: good drug compliance, poor drug compliance	
**Theme 2: Discharge planning process including ongoing clinical and functional assessment to facilitate the development of care plan and final discharge plan**	
**2a:** The four main dimensions for assessment should include medical health, physical, psychological and social functioning.	
**2b:** Care plan should be initiated within 24 h after admission.	Modified
**2c:** Three categories of discharge plans could be developed based on the complexity of patients and assessment of their needs:	
- Generic discharge plan suitable for simple case	
- Disease-based discharge plan suitable for complex cases when there are disease specific protocols	
- Non-disease specific, but tailored, discharge plan for complex cases identifying either by HARRPE or by assessment	
**2d:** Ongoing assessment/evaluation should be conducted throughout the episode of care to review and update the conditions of patients.	
**2e:** Effective and accessible IT systems for the accurate and timely communication of assessment and associated care planning information across clinical disciplines and settings should be developed and implemented to enhance care continuity (priority for high risk groups).	Modified
**Theme 3: Coordination of discharge - continuing and timely process from hospital stay to discharge**	
**3a:** A designated person e.g. a designated doctor, nurse, or allied health professional should be notionally responsible for ensuring that all aspects of discharge planning have been addressed by the time of discharge.	
**3b:** The role and responsibility of different healthcare professionals for the different tasks in the discharge planning process should be clarified.	Newly added
**3c:** Once the patient is identified to have complex care needs, the designated person should initiate discharge planning with a multidisciplinary approach.	
**3d:** Case conference should be considered as one of the options for high risk patients for better communication between team members in the multidisciplinary team and to enable seamless and timely transition from hospital to community.	Modified
**3e:** The suitability of discharge destination e.g. whether home or old-aged home, should be assessed to ascertain whether the support required is available.	
**3f:** Referral/arrangement for social support services should be initiated once the patient is assessed to have post discharge support need in the community.	
**3g:** Formal mechanisms for information transfer to community services providers for continuity of care should be established.	Modified
**3h:** Prompt provision of essential community equipment including walking aids, wheelchairs, low vision or hearing aids, safety alarm, urinal, blood pressure machines, glucometers, visual door etc. should be facilitated before discharge.	Modified
**3i:** Appropriate education and training should be provided to patients/carers to ensure that they understand how to use the equipment.	
**3j:** Appropriate information and education on medication management including side effects of medication should be provided to patients/carers before discharge.	
**Theme 4: Implementation of discharge**	
**4a:** Patients and/or carers should be engaged in the preparation of the discharge process.	
**4b:** Appropriate information on their illness should be given to the patients/carers to ensure that they could manage their ongoing care after discharge.	
**4c:** Patients/carers should be informed of any danger signals they should be aware of before discharge.	
**4d:** A specifically designed patient discharge summary including clinical diagnosis, follow-up and investigation appointments, medication and nursing care and instructions for allied health and social support services, should be given to patients/carers upon discharge.	Modified
**4e:** If the patient has complex care needs/disease specific problem, a contact information should be provided on who to contact if they are concerned about their condition or treatment after discharge.	
**4f:** Discharge summaries with necessary information should be issued to the facilities or care providers e.g. old aged homes within 48 h of discharge.	
**4g:** Discharge summaries with necessary information should be issued to the Hospital Authority outpatient and day care services within a week of discharge.	
**4h:** Timely transport arrangement for discharged patients should be made if necessary.	Modified
**4i:** Timely transport arrangements when attending outpatient appointments should be made if necessary.	
**4j:** A “Patient Checklist” should be completed before discharge to ensure that they understand the discharge plan and their needs are addressed.	Modified
**Theme 5: Post discharge follow up**	
**5a:** If the patient has complex care needs and is transferred from an acute hospital to a rehabilitation hospital, verbal communication via telephone or written information about the patient’s conditions should be made between the healthcare professionals in acute and rehabilitation hospitals.	-
**5b:** If the patient is referred to disease specific or special discharge programmes, person-to-person communication or written information about the patient’s conditions should be made between different parties.	

### Differences in degree of consensus by type of healthcare professions

Analyzing the degree of consensus by the type of healthcare professions, only the “clarity” aspect of the statement “*Q2d: Ongoing assessment/evaluation should be conducted throughout the episode of care.*” was found to be significantly different among the 5 healthcare professions (p-value <0.05). Medical social workers rated 4 which was a relatively lower rating compared to rating of 5 given by the other 4 healthcare professionals (doctors, nurses, physiotherapies and occupational therapists) to the “clarity” aspect of this statement. It might be due to the variation in healthcare professionals’ perspectives and involvement in the discharge planning. Furthermore, in-depth study to explore individual healthcare professionals’ perspectives and views is warranted.

## Discussion

Discharge planning is integral to good clinical care but is a variable process in most acute care settings especially in Hong Kong. A structured and coordinated system of hospital discharge is required if patients are to receive appropriate care and services in the community. Some developed countries such as Australia, US and UK had a relatively well-defined framework for discharge planning in hospitals. However, in Hong Kong, except for specific high risk groups who could be identified by clinical outcome score such as fall risk assessment, skin integrity and readmission risk algorithm [29] and those patients involved in the intervention programmes, there is no standardized discharge practice for other general patients in acute hospitals [6]. Moreover, there are many and very variable discharge programmes and process in different hospitals. The acute wards in hospitals, including Hong Kong, are very busy, and the median length of stay of a medical patient was around 3 days [30]. This rapid turnover of large number of patients with acute (and often undifferentiated) illness presents a great challenge for a discharge planning system. A structured, systematic and coordinated system of hospital discharge is required to facilitate the discharge process and ensure a smooth patient transition from hospital to community. Our study adopted a Delphi approach to validate and attest to the discharge planning framework developed in a 3-staged methodology in clarity, applicability and practicability for all the patients in hospitals. Apart from adding value to the existing research evidence, the findings can act as a reference, helping policymakers and hospital managers to facilitate the discharge planning process to improve the quality of care and decrease unnecessary hospital readmissions.

### Key components of discharge planning

Discharge is a complicated process involving different phases and aspects of care. Recognition of the components of an effective discharge can facilitate organizations in designing care delivery and orienting staff to discharge planning [31]. The literature highlighted that an efficient discharge required a provision of timely and informative risk screening for high risk patients, commencement or preparation of a discharge plan upon admission, timely notification of community providers [13,32]. Our findings from international literatures identified the key components of discharge planning under 5 major themes: (i) initial screening and assessment, (ii) discharge planning process including ongoing clinical and functional assessment to facilitate the development of care plan and final discharge plan, (iii) coordination of discharge including continuing and timely process from hospital stay to discharge, (iv) implementation of discharge focusing on patient readiness, post discharge service availability, and arrangement check before discharge, and (v) post discharge follow up. This framework provides a basis for developing more specific discharge planning protocol or care pathway for different type of patients in different settings. In addition, our 3-stage approach in developing a discharge policy framework involved statements on collaboration/communication between different type of healthcare professionals, patients, carers, and community service provision. Hedges pointed out that this component of collaboration was important to facilitate the timely discharge from hospital [32].

### Initial screening and assessment

The initial screening and assessment is important to differentiate patients with different risks and complexities in care needs for discharge planning. UK specified that discharge planning should be classified as simple or complex discharge at the point of patient admission [16]. With regard to the risk assessment tools, the validity of the statement on *“Using Hospital Admission Risk Reduction Program for the Elderly (HARRPE)* [HARRPE has been developed by the Hong Kong Hospital Authority for patients over the age of 60 on the basis of readmission risks which is a predictive modeling approach], *a screening tool developed by HA, to stratify patients with a higher risk of hospital readmission”* in our study was only 67% which was below the 75% level of consensus. The main concern of the participants on this statement was that there are a number of ways to identify patients who are likely to be high risk for readmission, and therefore HARRPE might not be the only or the best instrument. King’s Fund has conducted a literature review of the risk screening tools to develop a case-finding algorithm for high risk patients. Threshold approach, clinical knowledge, and predictive modeling are found to be three principal techniques in predicting risk [33]. HARRPE uses the clinical data of patients to model the risk of prediction of readmission in patients aged 60 or above. It includes the basic 13 specific risk factors: age, gender, living situation, functional status, cognitive status, behavior pattern, mobility, sensory deficit, number of previous admission, number of previous admission through Accident & Emergency Department, active medical disease, drugs, and need of referral. However, it does not contain the functional, cognitive status and mobility factor due to the unavailability of this data in the clinical management electronic system. Thus, our framework has proposed seven other items such as social support, care support, activity of daily living, functional status, mobility status, mental status, and fall history to supplement the tool and these risk screening items were well accepted by the participants in the study. These seven-item will then be used in our next stage of study to apply and pilot in a hospital to confirm its applicability and practicability.

### Timeliness of discharge planning

The literature highlighted the need for timely discharge planning in the discharge planning policy/guidelines [13,32]. To support timely and efficient discharge required provision of timely and informative risk screening for high risk patients, commencement or preparation of a discharge plan upon admission, timely notification of community providers including transport arrangement, and provision and transmission of a timely and informative discharge summary [11,32]. Collaboration between patient, carer, hospital staff and community services may well be required to facilitate the timely discharge from hospital [32]. The above component of timelines of discharge planning was included in most of the discharge policy/protocols. Timeliness of discharge planning, on the other hand, also served as the performance indicators for an effective discharge planning [11]. However, the criteria of timeliness e.g. within 24 h or 48 h varied among guidelines. NHS Trust in UK had set a timeframe of 24 h of admission to conduct a full nursing assessment, while another trust did not fix a timeframe, but required it to be commenced at the earliest possible stage [34]. The differences in the timeliness component were partly due to the setting and manpower constraint in the hospitals. In Hong Kong, we also faced the same problems in setting a timeframe in completing different tasks of discharge planning since the healthcare professionals were concerned about the issue of tight manpower and busy schedule in fulfilling the requirements due to the high turnover rate and caseload in the acute ward [6]. Our findings provided a discharge planning guideline on the timeframe of different milestones which was agreed by the local experts which takes into account its validity and applicability in the local context. These included (i) screening to be performed within 24 h after admission, (ii) care plan to be initiated within 24 h after admission, (iii) social support services to be initiated right after assessment, (iv) prompt provision of essential community equipment to be facilitated before discharge, (v) timely transportation to be arranged, (vi) discharge summary to be issued to (a) patients/care providers upon discharge, (b) health facilities or care providers such as old age home within 48 h of discharge, and (c) outpatient clinics within a week of discharge. These guidelines will also be piloted in the hospital to confirm it applicability and practicability.

### Role of different healthcare professionals in discharge planning

Regarding the concern of manpower management, the needs to clearly define roles and coordination of the team are also important components in a multidisciplinary approach [14,32]. Our study echoed this point by having a statement on *“The role and responsibility of different healthcare professionals for the different tasks in the discharge planning process should be clarified”* to be included in the framework after group discussion. This statement had a high level of validity and applicability (both 88%). Nearly all discharge planning policy/guidelines requires a designated person in coordinating discharge [13,14,32,34]. There are various models for the use of a single specialist to undertake discharge planning, for example, a discharge planner who has specialist knowledge and skills in discharge needs, community services and referrals; a patient care and/or admission coordinator who has specific responsibilities to improve communication and linkages between healthcare providers; or a case manager who focuses on the patient from admission to discharge, and to ensure earliest possible timely discharge. In Hong Kong, there is a Integrated Care Model (ICM) including a linked nurse to coordinate the inpatient services such as the formulation of care plan for post discharge care based on the comprehensive risk and needs assessment, and a case manager for the provision/coordination of the delivery of ambulatory and community health services. This ICM programme only applies to a small number of patients identified by the HARRPE screening system. The effect of discharge planning are generally quite mixed due to the diversity of the target patients served and the different ways of organizing a discharge plan [35].

### Communication in discharge planning

Our study findings suggested that communication between multidisciplinary team members, between hospital staff and community service providers, and between hospital staff and patients were vulnerable to breakdown. A formal communication mechanism, for example use of structured discharge summary and case conference was highlighted as formal communication options in the discharge planning guideline. Use of computer technology further facilitates the formal communication mechanism and it was highlighted in our findings but the confidentiality of data was a requirement [31]. Providing continuing education opportunities for hospital staff to acquire a better understanding of the multidisciplinary team members’ roles and community service provision might improve communication among multidisciplinary team and between hospital and community teams. In addition, open communication with and education for patients and family carers are crucial to successful and timely discharge planning [15,36]. Studies indicate that patient participation in discharge planning results in better health outcomes for patients and family carers following hospitalization and reduce avoidable readmissions [37,38].

### A standardized guideline for an effective discharge planning

The 3-staged process in the development of a discharge planning framework will provide a standardized guideline for an effective discharge planning to be applied in a local context. It addresses the current practice and the problem of a lack of standardized protocol for the discharge process [6]. The process also provides insight and reference on the conditions and conduct which will facilitate successful completion of a consensus framework by experts. Adopting a Delphi approach demonstrates the values of the method as a pre-test (before the clinical run) of the components and requirements of a discharge planning system taking into account the local context and system constraints, which would lead to improvements to its applicability and practicability.

To confirm the applicability and practicability of this consensus framework for discharge planning system, the third stage of process of development of the discharge planning framework is to apply and pilot the framework in a hospital setting to evaluate its feasibility, applicability and also impact in hospital including satisfaction from both the perspectives of staff and patients.

## Conclusions

A structured, systematic and coordinated system of hospital discharge system is required to facilitate the discharge process to ensure a smooth patient transition from the hospital to the community and improve patient health outcome in both clinical and social aspect. An effective discharge planning system benefits the hospital system with fewer unplanned readmissions, better quality of care and contributions to a better health care system. Our study is a 3-staged process to develop, pretest and pilot a discharge planning framework. This paper covers the second stage of the development of the framework, where we adopted a Delphi approach to pre-test its validity and attest to clarity, applicability and practicability of the requirement and components of discharge planning for all patients in the hospital system. In addition to adding the value to the existing research evidence, the findings provide a framework reference helping policymakers and hospital managers to facilitate the discharge planning process to improve the quality of care and decrease unnecessary hospital readmission.

## Competing interests

The authors declare that they have no competing interests.

## Authors’ contributions

All authors participated in the design of the project and the survey tool and carried out the study. CHKY performed the statistical analysis. The first draft of this article was composed by CHKY and ELYW and was revised critically by all authors. All authors read and approved the final version of the manuscript.

## Pre-publication history

The pre-publication history for this paper can be accessed here:

http://www.biomedcentral.com/1472-6963/12/396/prepub

## Supplementary Material

Additional file 1 Rating sheet for round 1 exercise at consensus discussion. Click here for file

## References

[B1] Anderson GF Steinberg EP Hospital readmissions in the Medicare population N Engl J Med 1984 311 1349 1353 10.1056/NEJM198411223112105 6436703

[B2] Parker SG Do current discharge arrangements from inpatient hospital care for the elderly reduce readmission rates, the length of inpatient stay or mortality, or improve health status? 2005 Copenhagen: WHO Regional Office for Europe

[B3] Jencks SF Williams MV Coleman EA Rehospitalizations among patients in the Medicare fee-for-service program N Engl J Med 2009 360 1418 1428 10.1056/NEJMsa0803563 19339721

[B4] Yam CHK Wong ELY Chan FWK Wong FYY Leung MCM Yeoh EK Measuring and preventing potentially avoidable hospital readmissions: a review of the literature HKMJ 2010 16 292 298 20890004

[B5] Wong ELY Cheung AWL Leung MCM Yam CHK Chan FWK Wong FYY Yeoh EK Unplanned readmission rates, length of hospital stay, mortality, and medical costs of ten common medical conditions: a retrospective analysis of Hong Kong hospital data BMC Health Serv Res 2011 11 149 10.1186/1472-6963-11-149 21679471 PMC3146405

[B6] Wong ELY Yam CHK Cheung AWL Leung MCM Chan FWK Wong FYY Yeoh EK Barriers to effective discharge planning: a qualitative study investigating the perspectives of frontline healthcare professionals BMC Health Serv Res 2011 11 242 10.1186/1472-6963-11-242 21955544 PMC3190337

[B7] Phillips CO Wright SM Kern DE Singa RM Shepperd S Rubin HR Comprehensive discharge planning with postdischarge support for older patients with congestive heart failure: a meta-analysis JAMA 2004 291 1358 1367 10.1001/jama.291.11.1358 15026403

[B8] Naylor MD Brooten D Campbell R Jacobsen BS Mezey MD Pauly MV Schwartz JS Comprehensive discharge planning and home follow-up of hospitalized elders: a randomized clinical trial JAMA 1999 281 613 620 10.1001/jama.281.7.613 10029122

[B9] Hyde CJ Robert IE Sinclair AJ The effects of supporting discharge from hospital to home in older people Age Ageing 2000 29 271 279 10.1093/ageing/29.3.271 10855913

[B10] Mamon J Steinwachs DM Fahey M Bone LR Oktay J Klein L Impact of hospital discharge planning on meeting patient needs after returning home Health Serv Res 1992 27 155 175 1317367 PMC1069871

[B11] Department of Human Services, Victoria Effective Discharge Strategy Background Paper: A Framework for Effective Discharge 1998 Available from: http://www.health.vic.gov.au/archive/archive2008/discharge/paper.htm

[B12] American Hospital Association Guidelines for Discharge Planning 1984 Chicago: AHA

[B13] Birmingham J Discharge planning: a collaboration between provider and payer case managers using Medicare’s Conditions of Participation Lippincotts Case Manag 2004 9 147 151 10.1097/00129234-200405000-00007 15252366

[B14] Summerton H Discharge planning: establishing an effective coordination team Br J Nurs 1998 7 1263 1267 9934033 10.12968/bjon.1998.7.20.5565

[B15] Katikireddi SV Cloud GC Planning a patient’s discharge from hospital BMJ 2008 337 a2694 10.1136/bmj.a2694 19074562

[B16] National Health Services Achieving timely ‘simple’ discharge from hospital - A toolkit for the multi-disciplinary team 2004 United Kindgom: Crown

[B17] Yam CHK Wong ELY Chan FWK Leung MCM Wong FYY Cheung AWL Yeoh EK Avoidable readmission in Hong Kong–system, clinician, patient or social factor? BMC Health Serv Res 2010 10 311 10.1186/1472-6963-10-311 21080970 PMC2993701

[B18] LeClerc M Wells DL Process evaluation of an integrated model of discharge planning Can J Nurs Leadersh 2001 14 19 26 15487300 10.12927/cjnl.2001.19120

[B19] Wells DL Martin DK Moorhouse A Craig D Foley JM An integrated model of discharge planning for acutely-ill elderly patients Can J Nurs Leadersh 1999 12 6 12 11094933 10.12927/cjnl.1999.19079

[B20] Linstone HA Turoff M The Delphi method: techniques and applications 1975 Reading: Addison-Wesley Pub. Co.

[B21] Michael A Erio Z Gazing Into the Oracle: The Delphi Method and Its Application to Social Policy and Public Health 1996 London: Kingsley Publishers

[B22] Fiore JF Jr Bialocerkowski A Browning L Faragher IG Denehy L Criteria to determine readiness for hospital discharge following colorectal surgery: an international consensus using the Delphi technique Dis Colon Rectum 2012 55 416 423 10.1097/DCR.0b013e318244a8f2 22426265

[B23] Atwal A Caldwell K Profiting from consensus methods in occupational therapy: using a delphi study to achieve consensus on multiprofessional discharge planning Br J Occup Ther 2003 66 6 65 70

[B24] Yam CHK Wong ELY Cheung AWL Chan FWK Wong FYY Yeoh EK Patient’s engagement in discharge planning process and their post discharge care need -“Finding from a Questionnaire Survey” Proceedings of the Hong Kong Hospital Authority Convention 2012: 7–8 May 2012 Hong Kong 2012 Hong Kong: Hong Kong Hospital Authority

[B25] Keeney S Hasson F McKenna H Consulting the oracle: ten lessons from using the Delphi technique in nursing research J Adv Nurs 2006 53 205 212 10.1111/j.1365-2648.2006.03716.x 16422719

[B26] Hasson F Keeney S McKenna H Research guidelines for the Delphi survey technique J Adv Nurs 2000 32 1008 1015 11095242

[B27] Ludwig B Predicting the future: have you considered using the Delphi methodology? J Ext 1997 35 1 4

[B28] McBride AJ Pates R Ramadan R McGowan C Delphi survey of experts’ opinions on strategies used by community pharmacists to reduce over-the-counter drug misuse Addiction 2003 98 487 497 10.1046/j.1360-0443.2003.00345.x 12653818

[B29] Chan S Kwong P Kong B To K Ho J Wong C Improving Health of High Risk Elderly in the Community - the HARRPE Proceedings of the Symposium on Community Engagement III “Creating a Synergy for Community Health” 2008; Hong Kong 2008 Hong Kong: Hong Kong Hospital Authority

[B30] Authority H Hospital Authority Statistical Report 2009–2010 2010 Hong Kong: Hospital Authority

[B31] Bull MJ Roberts J Components of a proper hospital discharge for elders J Adv Nurs 2001 35 571 581 10.1046/j.1365-2648.2001.01873.x 11529957

[B32] Hedges G Grimmer K Moss J Falco J Performance indicators for discharge planning: a focused review of the literature Aust J Adv Nurs 1999 16 20 28 10603768

[B33] Curry N Billings J Darin B Dixon J Williams M Wennberg D Predictive risk project - Literature Review 2005 United Kingdom Available from: http://www.kingsfund.org.uk/sites/files/kf/field/field_document/predictive-risk-literature-review-june2005.pdf

[B34] McHale SA Implementation of a patient discharge policy Prof Nurse 1995 10 590 592 7604059

[B35] Shepperd S McGlaran J Philips Com Kannin NA Clemson LM McCluskey A Discharge planning from hospital to home (Review) The Cochrane Collaboration 2010 1 1 75 10.1002/14651858.CD000313.pub3 20091507

[B36] Carroll A Dowling M Discharge planning: communication, education and patient participation Br J Nurs 2007 16 882 886 17851351 10.12968/bjon.2007.16.14.24328

[B37] Jewell S Elderly patients’ participation in discharge decision making: 2 Br J Nurs 1996 5 1065 1071 8918767 10.12968/bjon.1996.5.17.1065

[B38] Bull MJ Hansen HE Gross CR A professional-patient partnership model of discharge planning with elders hospitalized with heart failure Appl Nurs Res 2000 13 19 28 10.1016/S0897-1897(00)80015-4 10701280

