# Supplementary material for: Framework and components for effective discharge planning system: a delphi methodology
Source: BMC Health Serv Res. 2012 Nov 14;12:396. doi: 10.1186/1472-6963-12-396 (PMC3508885; doi:10.1186/1472-6963-12-396)
Supplement: Additional file 1 — Rating sheet for round 1 exercise at consensus discussion. [file 1472-6963-12-396-S1.doc]

| Round 1 Rating | Name: ________________________________ | Subject Code: __________ |
| --- | --- | --- |

Please circle the appropriate rating of (i) clarity, (ii) validity and (iii) applicability of each statement, and comment/ feedback on how to improve the statements if needed.

**Theme 1: Initial screening**

|  | **Clarity** | **Validity** | **Applicability** | **Comments** |
| --- | --- | --- | --- | --- |
|  | **Low Average High** | **Low Average High** | **Low Average High** | **In particular on rating 1-3** |
| 1. An initial risk screening should be performed within 24 hours after admission to identify those patients with high risk of admission and have complex discharge planning, required to provide ongoing care and additional support after leaving hospital. 2. HARRPE (Hospital Admissions Risk Reduction Program for the elderly), a screening tool developed by HA, could be used to stratify those elderly aged 60 or above with a higher risk of hospital readmission. 3. A patient with score of above 0.2 is considered as high risk and requires a complex discharge arrangement. | **1 2 3 4 5**  **1 2 3 4 5**  **1 2 3 4 5** | **1 2 3 4 5**  **1 2 3 4 5**  **1 2 3 4 5** | **1 2 3 4 5**  **1 2 3 4 5**  **1 2 3 4 5** |  |

**Theme 1: Initial screening (Cont’d)**

|  | **Clarity** | **Validity** | **Applicability** | **Comments** |
| --- | --- | --- | --- | --- |
|  | **Low Average High** | **Low Average High** | **Low Average High** | **In particular on rating 1-3** |
| The following items should be included in the initial assessment for all patients to serve as flags to trigger discharge planning as appropriate:   1. Social support – living alone, day time alone, night time alone, with maid, with spouse, with children, with grandchildren, with others. 2. Care support – Yes (by spouse, son, daughter-in-law, daughter, son-in-law, grandchildren, maid, others), No 3. Any change of ADL: ADL Barthel Index before admission, and on admission (declining ADL index) | **1 2 3 4 5**  **1 2 3 4 5**  **1 2 3 4 5** | **1 2 3 4 5**  **1 2 3 4 5**  **1 2 3 4 5** | **1 2 3 4 5**  **1 2 3 4 5**  **1 2 3 4 5** |  |

**Theme 1: Initial screening (Cont’d)**

|  | **Clarity** | **Validity** | **Applicability** | | **Comments** |
| --- | --- | --- | --- | --- | --- |
|  | **Low Average High** | **Low Average High** | **Low Average High** | | **In particular on rating 1-3** |
| The following items should be included in the initial assessment for all patients to serve as flags to trigger discharge planning as appropriate:   1. Functional ambulatory category (modified): lyer, sitter, dependent walker, assisted walker, supervised walker, indoor walker, outdoor walker (independent, assisted with carer, assisted with equipment) 2. History of fall risk for the past one year: No history of fall, history of fall = 1, recurrent falls, present to medical attention for fall, both risk factors are present 3. Mental state: normal, disorientated, disturbed, poor memory, not communicate 4. Medications: good drug compliance, poor drug compliance | **1 2 3 4 5**  **1 2 3 4 5**  **1 2 3 4 5**  **1 2 3 4 5** | **1 2 3 4 5**  **1 2 3 4 5**  **1 2 3 4 5**  **1 2 3 4 5** | **1 2 3 4 5**  **1 2 3 4 5**  **1 2 3 4 5**  **1 2 3 4 5** | |  |
| Round 1 Rating | Name: ________________________________ | | | Subject Code: __________ | |

Please circle the appropriate rating of (i) clarity, (ii) validity and (iii) applicability of each statement, and comment/ feedback on how to improve the statements if needed.

**Theme 2: Discharge planning process: including ongoing clinical and functional assessment to facilitate the development of care plan and final discharge plan**

|  | **Clarity** | **Validity** | **Applicability** | **Comments** |
| --- | --- | --- | --- | --- |
|  | **Low Average High** | **Low Average High** | **Low Average High** | **In particular on rating 1-3** |
| 1. The four main dimensions for assessment should include medical health, physical, psychological and social functioning. 2. Care plan should be performed within 24 hours after admission. | **1 2 3 4 5**  **1 2 3 4 5** | **1 2 3 4 5**  **1 2 3 4 5** | **1 2 3 4 5**  **1 2 3 4 5** |  |

**Theme 2: Discharge planning process: including ongoing clinical and functional assessment to facilitate the development of care plan and final discharge plan (Cont’d)**

|  | **Clarity** | **Validity** | **Applicability** | **Comments** |
| --- | --- | --- | --- | --- |
|  | **Low Average High** | **Low Average High** | **Low Average High** | **In particular on rating 1-3** |
| 1. Three categories of discharge plans could be developed based on the complexity of patients and assessment of their needs:    - Generic discharge plan suitable for simple cases    - Disease-based discharge plan suitable for complex cases when there are disease specific protocols    - Non-disease specific, but tailored, discharge plan for complex cases identifying either by HARRPE or by assessment 2. Ongoing assessment /evaluation should be conducted throughout the episode of care to review and update the conditions of patients. | **1 2 3 4 5**  **1 2 3 4 5** | **1 2 3 4 5**  **1 2 3 4 5** | **1 2 3 4 5**  **1 2 3 4 5** |  |

**Theme 2: Discharge planning process: including ongoing clinical and functional assessment to facilitate the development of care plan and final discharge plan (Cont’d)**

|  | **Clarity** | **Validity** | **Applicability** | **Comments** |
| --- | --- | --- | --- | --- |
|  | **Low Average High** | **Low Average High** | **Low Average High** | **In particular on rating 1-3** |
| 1. Systems for the accurate and timely communication of assessment and associated care planning information across clinical disciplines and settings should be developed and implemented to enhance care continuity. | **1 2 3 4 5** | **1 2 3 4 5** | **1 2 3 4 5** |  |

| Round 1 Rating | Name: ________________________________ | Subject Code: __________ |
| --- | --- | --- |

Please circle the appropriate rating of (i) clarity, (ii) validity and (iii) applicability of each statement, and comment/ feedback on how to improve the statements if needed.

**Theme 3: Coordination of discharge: continuing and timely process from hospital stay to discharge**

|  | **Clarity** | **Validity** | **Applicability** | **Comments** |
| --- | --- | --- | --- | --- |
|  | **Low Average High** | **Low Average High** | **Low Average High** | **In particular on rating 1-3** |
| 1. A designated person e.g. a designated doctor, nurse, or allied health professional should be notionally responsible for ensuring that all aspects of discharge planning have been addressed by the time of discharge. 2. Once the patient is identified to have complex care needs, the designated person should initiate discharge planning with a multidisciplinary approach. 3. Case conference should be considered for high risk patients for better communication between team members in the multidisciplinary team and to enable seamless and timely transition from hospital to community. | **1 2 3 4 5**  **1 2 3 4 5**  **1 2 3 4 5** | **1 2 3 4 5**  **1 2 3 4 5**  **1 2 3 4 5** | **1 2 3 4 5**  **1 2 3 4 5**  **1 2 3 4 5** |  |

**Theme 3: Coordination of discharge: continuing and timely process from hospital stay to discharge (Cont’d)**

|  | **Clarity** | **Validity** | **Applicability** | **Comments** |
| --- | --- | --- | --- | --- |
|  | **Low Average High** | **Low Average High** | **Low Average High** | **In particular on rating 1-3** |
| 1. The suitability of discharge destination e.g. whether home or old-aged home, should be assessed to ascertain whether the support required is available. 2. Referral/ arrangement for social support services should be initiated once the patient is assessed to have post discharge support need in the community. 3. Formal mechanisms for information transfer across clinical and social settings e.g. through discharge summary should be adopted rather than solely relying on informal communication between health and social professionals. | **1 2 3 4 5**  **1 2 3 4 5**  **1 2 3 4 5** | **1 2 3 4 5**  **1 2 3 4 5**  **1 2 3 4 5** | **1 2 3 4 5**  **1 2 3 4 5**  **1 2 3 4 5** |  |

**Theme 3: Coordination of discharge: continuing and timely process from hospital stay to discharge (Cont’d)**

|  | **Clarity** | **Validity** | **Applicability** | **Comments** |
| --- | --- | --- | --- | --- |
|  | **Low Average High** | **Low Average High** | **Low Average High** | **In particular on rating 1-3** |
| 1. Prompt provision of all community equipment including walking aids, wheelchairs, low vision or hearing aids, safety alarm, urinal, blood pressure machines, glucometers, visual door etc. should be ensured before discharge. 2. Appropriate education and training should be provided to patients / carers to ensure that they understand how to use the equipment. 3. Appropriate information and education on medication management including side effects of medication should be provided to patients/ carers before discharge. | **1 2 3 4 5**  **1 2 3 4 5**  **1 2 3 4 5** | **1 2 3 4 5**  **1 2 3 4 5**  **1 2 3 4 5** | **1 2 3 4 5**  **1 2 3 4 5**  **1 2 3 4 5** |  |

| Round 1 Rating | Name: ________________________________ | Subject Code: __________ |
| --- | --- | --- |

Please circle the appropriate rating of (i) clarity, (ii) validity and (iii) applicability of each statement, and comment/ feedback on how to improve the statements if needed.

**Theme 4: Implementation of discharge**

|  | **Clarity** | **Validity** | **Applicability** | **Comments** |
| --- | --- | --- | --- | --- |
|  | **Low Average High** | **Low Average High** | **Low Average High** | **In particular on rating 1-3** |
| 1. Patients and/ or carers should be engaged in the preparation of the discharge process. 2. Appropriate information on their illness should be given to the patients/ carers to ensure that they could manage their ongoing care after discharge. 3. Patients/ carers should be informed of any danger signals they should be aware of before discharge. 4. A patient copy of discharge summary and/ or nursing discharge summary should be given to patients/ carers on the date of discharge. | **1 2 3 4 5**  **1 2 3 4 5**  **1 2 3 4 5**  **1 2 3 4 5** | **1 2 3 4 5**  **1 2 3 4 5**  **1 2 3 4 5**  **1 2 3 4 5** | **1 2 3 4 5**  **1 2 3 4 5**  **1 2 3 4 5**  **1 2 3 4 5** |  |

**Theme 4: Implementation of discharge (Cont’d)**

|  | **Clarity** | **Validity** | **Applicability** | **Comments** |
| --- | --- | --- | --- | --- |
|  | **Low Average High** | **Low Average High** | **Low Average High** | **In particular on rating 1-3** |
| 1. If the patient has complex care needs/ disease specific problem, a contact information should be provided on who to contact if they are concerned about their condition or treatment after discharge. 2. Discharge summaries with necessary information should be issued to the facilities or care providers e.g. old aged homes within 48 hours of discharge. 3. Discharge summaries with necessary information should be issued to the Hospital Authority outpatient and day care services within a week of discharge. 4. When transport is to be used, this should be booked at least 24 hours, where feasible, in advance of discharge. | **1 2 3 4 5**  **1 2 3 4 5**  **1 2 3 4 5**  **1 2 3 4 5** | **1 2 3 4 5**  **1 2 3 4 5**  **1 2 3 4 5**  **1 2 3 4 5** | **1 2 3 4 5**  **1 2 3 4 5**  **1 2 3 4 5**  **1 2 3 4 5** |  |

**Theme 4: Implementation of discharge (Cont’d)**

|  | **Clarity** | **Validity** | **Applicability** | **Comments** |
| --- | --- | --- | --- | --- |
|  | **Low Average High** | **Low Average High** | **Low Average High** | **In particular on rating 1-3** |
| 1. Timely transport arrangements when attending outpatient appointments should be made if necessary. 2. A “Patient Checklist” should be completed by the patient or carers before discharge to ensure that they understand the discharge plan and their needs are addressed. | **1 2 3 4 5**  **1 2 3 4 5** | **1 2 3 4 5**  **1 2 3 4 5** | **1 2 3 4 5**  **1 2 3 4 5** |  |

| Round 1 Rating | Name: ________________________________ | Subject Code: __________ |
| --- | --- | --- |

Please circle the appropriate rating of (i) clarity, (ii) validity and (iii) applicability of each statement, and comment/ feedback on how to improve the statements if needed.

**Theme 5: Post discharge follow up**

|  | **Clarity** | **Validity** | **Applicability** | **Comments** |
| --- | --- | --- | --- | --- |
|  | **Low Average High** | **Low Average High** | **Low Average High** | **In particular on rating 1-3** |
| 1. If the patient has complex care needs and is transferred from an acute hospital to a rehabilitation hospital, verbal communication via telephone or written information about the patient’s conditions should be made between the healthcare professionals in acute and rehabilitation hospitals. 2. If the patient is referred to disease specific or special discharge programmes, person-to-person communication or written information about the patient’s conditions should be made between different parties. | **1 2 3 4 5**  **1 2 3 4 5** | **1 2 3 4 5**  **1 2 3 4 5** | **1 2 3 4 5**  **1 2 3 4 5** |  |
